# Structure-Function analysis of the CTLA-4 interaction with PP2A

**DOI:** 10.1186/1471-2172-10-23

**Published:** 2009-04-30

**Authors:** Wendy A Teft, Thu A Chau, Joaquín Madrenas

**Affiliations:** 1 FOCIS Centre for Clinical Immunology and Immunotherapeutics, Robarts Research Institute, The University of Western Ontario, London, Ontario, N6A 5K8, Canada; 2 Departments of Microbiology and Immunology, The University of Western Ontario, London, Ontario, N6A 5K8, Canada; 3 Department of Medicine, The University of Western Ontario, London, Ontario, N6A 5K8, Canada; 4 Robarts Research Institute, PO Box 5015, 100 Perth Drive, London ON, N6A 5K8, Canada

## Abstract

**Background:**

CTLA-4 functions primarily as an inhibitor of T cell activation. There are several candidate explanations as to how CTLA-4 modulates T cell responses, but the exact mechanism remains undefined. The tail of CTLA-4 does not have any intrinsic enzymatic activity but is able to associate with several signaling molecules including the serine/threonine phosphatase PP2A. PP2A is a heterotrimeric molecule comprised of a regulatory B subunit associated with a core dimer of a scaffolding (A) and a catalytic (C) subunit.

**Results:**

Here, we performed an analysis of the human CTLA-4 interface interacting with PP2A. We show that PP2A interacts with the cytoplasmic tail of CTLA-4 in two different sites, one on the lysine rich motif, and the other on the tyrosine residue located at position 182 (but not the tyrosine 165 of the YVKM motif). Although the interaction between CTLA-4 and PP2A was not required for inhibition of T cell responses, it was important for T cell activation by inverse agonists of CTLA-4. Such an interaction was functionally relevant because the inverse agonists induced IL-2 production in an okadaic acid-dependent manner.

**Conclusion:**

Our studies demonstrate that PP2A interacts with the cytoplasmic tail of human CTLA-4 through two motifs, the lysine rich motif centered at lysine 155 and the tyrosine residue 182. This interaction and the phosphatase activity of PP2A are important for CTLA-4-mediated T cell activation.

## Background

Cytotoxic T lymphocyte associated antigen-4 (CTLA-4, CD152) is an activation-induced glycoprotein of the Immunoglobulin superfamily, whose primary function is to down-regulate T cell responses [1-4]. CTLA-4 shares its two known endogenous ligands, the B7 molecules B7.1 (CD80) and B7.2 (CD86), with the costimulatory receptor CD28 [5-7]. Several mechanisms, including antagonism of CD28-dependent costimulation and direct negative signaling have been documented to explain the inhibitory capacity of CTLA-4 [8]. Since the cytoplasmic tail of CTLA-4 lacks intrinsic enzymatic activity, the delivery of such a negative signal is likely provided through the association of CTLA-4 with key signaling molecules [4].

CTLA-4 has been shown independently by two groups to associate with the serine/threonine phosphatase PP2A [9,10]. PP2A is a heterotrimeric holoenzyme which is comprised of a regulatory B subunit associated with a core dimer of a scaffolding A subunit (PP2AA) and a catalytic C subunit (PP2AC) [11]. PP2A accounts for close to 1% of all cellular proteins and provides the majority of serine/threonine phosphate activity within eukaryotic cells [12]. Using recombinant proteins, it has been reported that PP2AA interacts with the lysine rich motif located in the juxtamembrane region of the cytoplasmic tail of human CTLA-4, while the C subunit is thought to interact with the tyrosine residue in the YVKM motif located at position 165 [9,10]. However, it is currently unknown whether some of these associations occur *in vivo *in T cells and if so what the functional consequences are.

We have previously reported that PP2A may regulate the ability of CTLA-4 to act as an inhibitor. Newly synthesized CTLA-4 becomes associated with PP2AA and remains associated when expressed on the cell surface, effectively blocking its inhibitory function [10]. Following TCR:CTLA-4 co-ligation, where CTLA-4 engages B7 molecules expressed on antigen-presenting cells (APCs), PP2A is phosphorylated and dissociates from CTLA-4, and this dissociation correlates with the attenuation of T cell activation [10]. Additionally, CTLA-4-dependent inhibition of Akt, a downstream target of PP2A, is sensitive to the PP2A inhibitor okadaic acid, implying that PP2A plays an important role in CTLA-4-mediated T cell inactivation [13].

Under unique circumstances, some recombinant ligands of CTLA-4 can act as inverse agonists making CTLA-4 capable of activating T cells by itself, independent of TCR or CD28 ligation [14,15]. We have recently shown that soluble B7.1 Ig or 24:26, a bispecific, in-tandem single-chain Fv (ScFv) against human CTLA-4, function as inverse agonists of CTLA-4 resulting in the activation of primary human T cells and T cell lines. Such an inverse agonist activity correlates with the ability to induce the formation of a unique dimer-based CTLA-4 oligomer that signals through its cytoplasmic tail [15]. Under these conditions of ligation, we have observed an increased association between PP2A and CTLA-4 suggesting that CTLA-4 may also induce T cell activation in a PP2A-dependent manner [14].

As suggested by Rudd, the role of PP2A in CTLA-4 function needs clarification [16]. Here, we started to address this issue by showing for the first time that the association between CTLA-4 and PP2A occurs in primary human T cells, suggesting that this interaction is physiologically relevant. Furthermore, we characterized the CTLA-4 interface interacting with PP2A using a panel of stably transfected Jurkat T cells expressing either wildtype (WT) CTLA-4 or CTLA-4 molecules mutated at various residues within the cytoplasmic domain. In this way, we eliminated any confounding effects as Jurkat T cells do not express endogenous CTLA-4 [17]. Our results confirm the importance of the lysine rich motif for the association of PP2AA. However, contrary to previous studies, we report that not the first but the second tyrosine residue located at position 182 of human CTLA-4 is important for the binding of PP2AC to CTLA-4. Functionally, an increase in the association of PP2A to CTLA-4 was observed under conditions of inverse agonist ligation of CTLA-4 molecules with the exception of those mutated at the lysine residues. Such an increase correlated with the ability of CTLA-4 to induce T cell activation, and was dependent on the enzymatic activity of PP2A.

## Results

### CTLA-4 interacts with PP2A in primary human T cells

**Figure 1:**
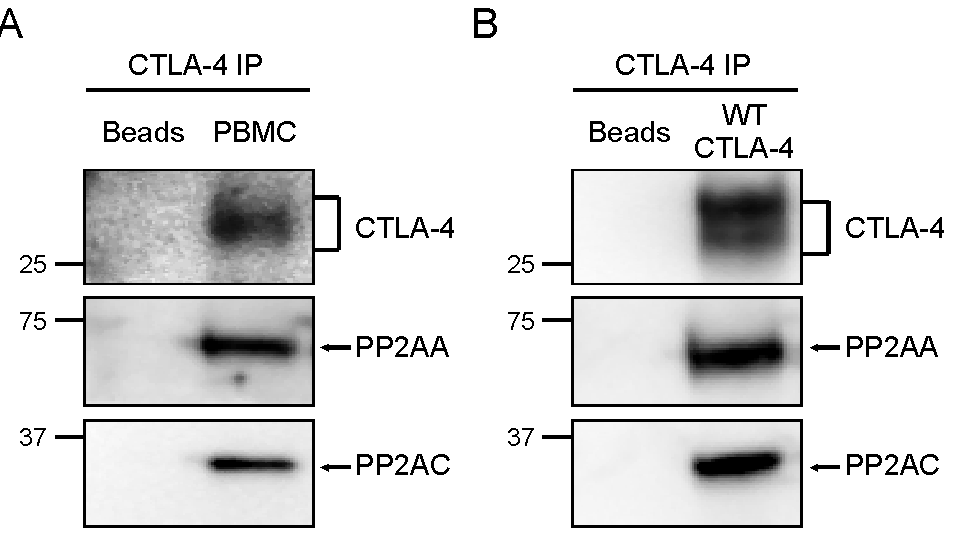
**CTLA-4 interacts with PP2A in primary human T cells and Jurkat T cells**. A) PBMC were isolated from healthy donors and cultured in the presence of PMA (1 ng/ml) and ionomycin (100 ng/ml) for 72 h. The cells were washed extensively and rested for 24 h in fresh media. Cell lysates were immunoprecipitated using anti-CTLA-4 Abs and immunoblotted for CTLA-4, PP2AA and PP2AC. B) Jurkat T cells stably transfected with WT CTLA-4 were cultured overnight in the presence of doxycycline (1 μg/ml) to induce the expression of CTLA-4. Cell lysates were immunoprecipitated and immunoblotted as in A).

The interaction between CTLA-4 and PP2A has previously been shown by yeast two hybrid analysis and transfected cell lines [9,10]. However, this association has not been confirmed in primary T cells. Thus, we first explored this interaction using peripheral blood mononuclear cells (PBMC) isolated from healthy donors and cultured in the presence of PMA and ionomycin for 72 h to induce the expression of CTLA-4. The cells were washed and rested in fresh media for an additional 24 h before cell lysates were immunoprecipitated using anti-CTLA-4 antibodies (Abs) and immunoblotted for CTLA-4, PP2AA and PP2AC. As shown in figure 1A, both the scaffolding unit and the catalytic unit of PP2A (PP2AA and PP2AC, respectively), which together form the core PP2A enzyme, co-precipitated with CTLA-4 in primary T cells.

To gain insight into the interaction between CTLA-4 and PP2A we needed to use a feasible model system in which we could express human wildtype (WT) or mutant CTLA-4 molecules in the absence of endogenous CTLA-4 and assay for their association with PP2A. Therefore, we examined the CTLA-4:PP2A association in Jurkat T cells that had been stably transfected with WT CTLA-4 under the control of a doxycycline inducible promoter. We have previously reported that these cells lack expression of endogenous CTLA-4, thus eliminating any masking effect on the results [17]. After overnight culture in the presence of doxycycline to induce the expression of CTLA-4, lysates from these transfected Jurkat T cells were prepared and subsequently immunoprecipitated using anti-CTLA-4 Abs (Figure 1B). As seen in PBMC, WT CTLA-4 associated with PP2AA and PP2AC, indicating that this model system is appropriate to perform a structure:function analysis of the CTLA-4 interface interacting with PP2A.

### Surface expression of WT and mutant CTLA-4 molecules

**Figure 2:**
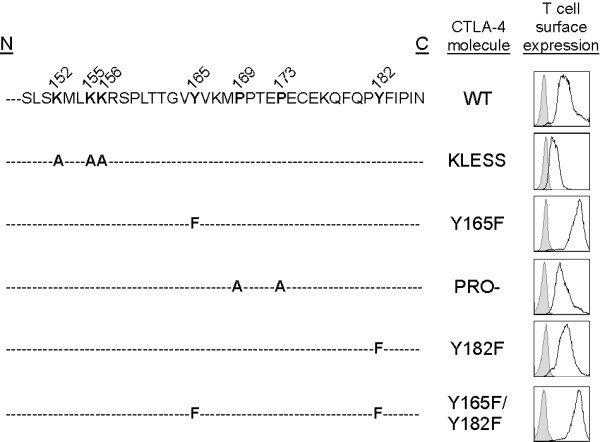
**Sequence and surface expression CTLA-4 molecules used in these studies**. The WT sequence of the intracellular tail of CTLA-4 is shown in full with key residues targeted for mutation depicted in bold. Lysine residues 152, 155 and 156 were changed to alanine residues to generate KLESS CTLA-4. Tyrosine residues located at positions 165 or 182 have been converted to phenylalanine to create Y165F CTLA-4 and Y182F CTLA-4, respectively. The double mutant Y165F/Y182F CTLA-4 contains mutations at both tyrosine residues. PRO- CTLA-4 contains mutations of proline residues 169 and 173 to alanine residues. Stably transfected Jurkat T cell lines have been generated for each of these CTLA-4 variants. Cells were induced overnight with doxycycline (1 μg/ml) and the surface expression of CTLA-4 was measured by flow cytometry (black line, CTLA-4; shaded profile, isotype-matched Ab).

To dissect the interaction between CTLA-4 and PP2A we used a panel of doxycycline-inducible Jurkat T cells stably transfected with WT CTLA-4 or CTLA-4 molecules containing mutations within the cytoplasmic domain. The intracellular tail of CTLA-4 is 100% conserved among mammalian species suggesting that this domain is important for the function of CTLA-4 [18]. Although the intracellular portion of CTLA-4 does not have any intrinsic enzymatic activity, it contains several motifs that may be important for its interaction with key signaling molecules [4]. Thus, we examined CTLA-4 molecules containing mutations at each of these putative motifs. These included the lysine rich motif (KLESS) located in the juxtamembrane region of the tail and the tyrosine residue located at position 165 (Y165F), both sites previously claimed to be important for the interaction with PP2A [9,10]. CTLA-4 molecules with mutations at the second tyrosine residue (Y182F), at both tyrosine residues (Y165F/Y182F), and at the proline rich domain (PRO-) located at residues 169–173 were also used. These Jurkat T cell lines were cultured overnight with doxycycline to induce the expression CTLA-4. The surface expression of each CTLA-4 variant was measured by flow cytometry (Figure 2). Similar levels of surface expression were observed for cells expressing WT (mean fluorescence intensity (MFI), 691), PRO- (MFI, 330) and Y182F (MFI, 432) CTLA-4 molecules. Lower levels of surface expression were detected for KLESS CTLA-4 (MFI, 43), while an increase in the level of CTLA-4 on the surface was observed for cells expressing molecules mutated at position Y165 (both Y165F (MFI, 1772), and Y165F/Y182F(MFI, 1757)), likely owing to their inability to be effectively internalized by AP-2 [19-21].

### PP2A interacts with CTLA-4 at lysines 152, 155 and 156 and tyrosine 182

**Figure 3:**
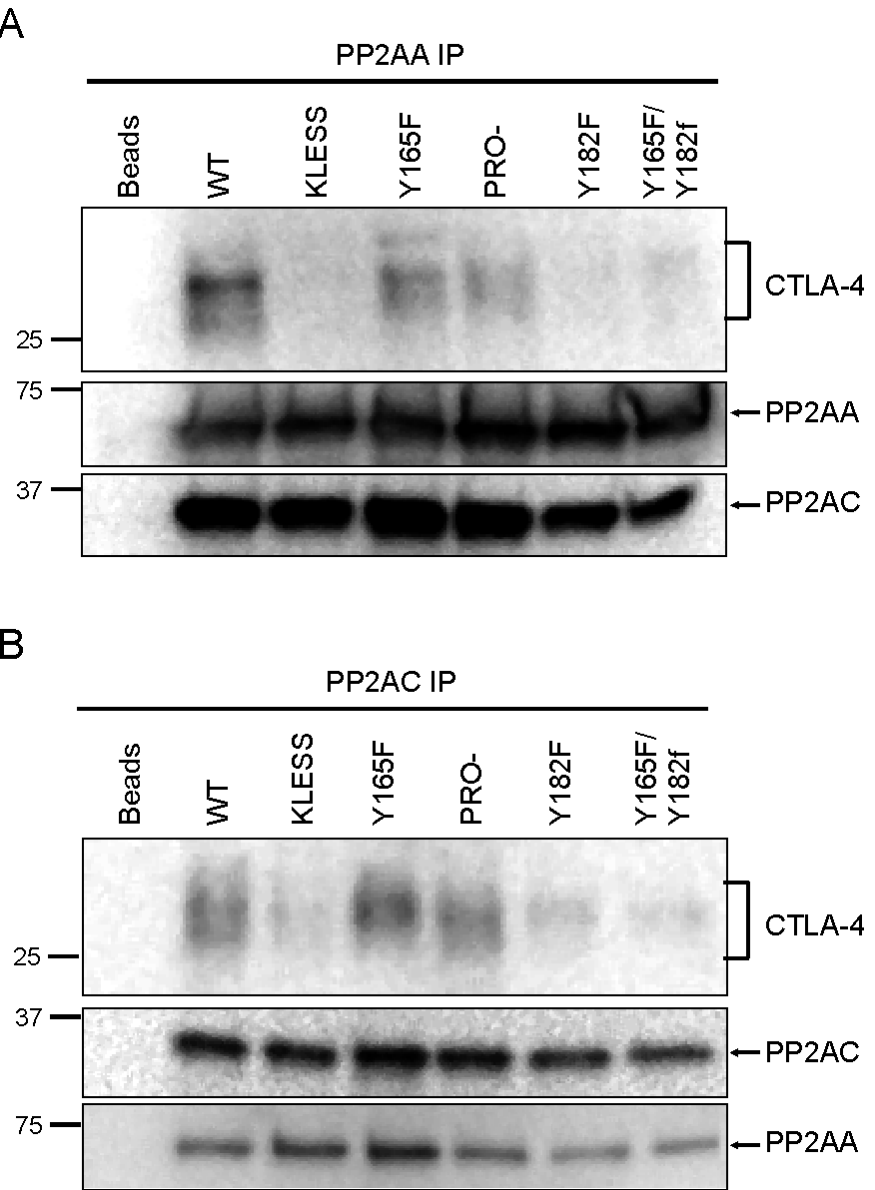
**PP2A interacts with CTLA-4 through the lysine rich domain and tyrosine 182**. A) Association of CTLA-4 with the A subunit of PP2A. Jurkat T cell lines stably transfected with CTLA-4 variants were cultured overnight in the presence of doxycycline (1 μg/ml) to induce the expression of CTLA-4. Cell lysates were prepared and used for immunoprecipitation of PP2AA, followed by immunoblotting for CTLA-4, PP2AA and PP2AC. B) Association of CTLA-4 with the C subunit of PP2A. Jurkat cells were cultured and lysed as in A) and used for immunoprecipitation of PP2AC, followed by blotting for CTLA-4, PP2AA and PP2AC. Beads, immunoprecipitating Ab without cell lysate. Blots are representative of at least 3 independent experiments.

PP2A is a heterotrimeric complex comprised of a core dimer consisting of the A and C subunits and a regulatory B subunit thought to provide substrate specificity and/or localization within the cell [11]. Previous reports have demonstrated that CTLA-4 can interact with both the A and C subunits of PP2A [9,10]. Using our panel of CTLA-4 mutants we performed a structural analysis of the association of both PP2AA and PP2AC subunits with CTLA-4. To do this, we induced the expression of the different CTLA-4 mutants, immunoprecipitated PP2A using anti-PP2AA or anti-PP2AC Abs and immunoblotted for CTLA-4 (Figure 3). Under these conditions the amount of immunoprecipitated PP2A represents approximately 1.5% of total PP2A. Equal loading was confirmed by blotting for the immunoprecipitating Ab. As expected, we observed co-precipitation of both PP2AA and PP2AC with WT CTLA-4. On average approximately 2% of total CTLA-4 immunoprecipitated with PP2A. As it is the surface pool of CTLA-4 (representing approximately 10% of total CTLA-4) that associates with PP2A, we estimate that approximately 20% of surface CTLA-4 is associated with PP2A. Additionally, CTLA-4 variants mutated at the proline rich motif were able to co-precipitate with PP2A at similar levels compared to WT CTLA-4, suggesting that these residues are not essential in the CTLA-4:PP2A interaction.

As expected [10], KLESS CTLA-4 molecules failed to interact with PP2AA (Figure 3A). The association between PP2AC and KLESS CTLA-4 was also significantly diminished (Figure 3B). This result correlated with the observation that *in vivo *PP2AA is always found in association with PP2AC, suggesting that intact binding sites for both subunits may be required to establish a stable interaction between PP2A and CTLA-4. Surprisingly, we found that Y165F CTLA-4 associated with PP2AA and PP2AC at similar levels compared to WT CTLA-4, implying that this residue may not be the main putative binding site for the catalytic subunit of PP2A as previously reported [9]. Alternatively, the second tyrosine residue in the cytoplasmic tail (Y182) may provide a non-canonical binding site for PP2AC in the absence of Y165 because mutation of tyrosine 182 prevented the interaction between CTLA-4 and PP2A (Figure 3A, B). Similarly, the double tyrosine mutant, Y165F/Y182F failed to interact *in vivo *with PP2A, further corroborating the key role of the Y182 as the putative primary binding site for the catalytic subunit of PP2A.

### Effect of mutations in the intracellular domain of CTLA-4 on its inhibitory function

**Figure 4:**
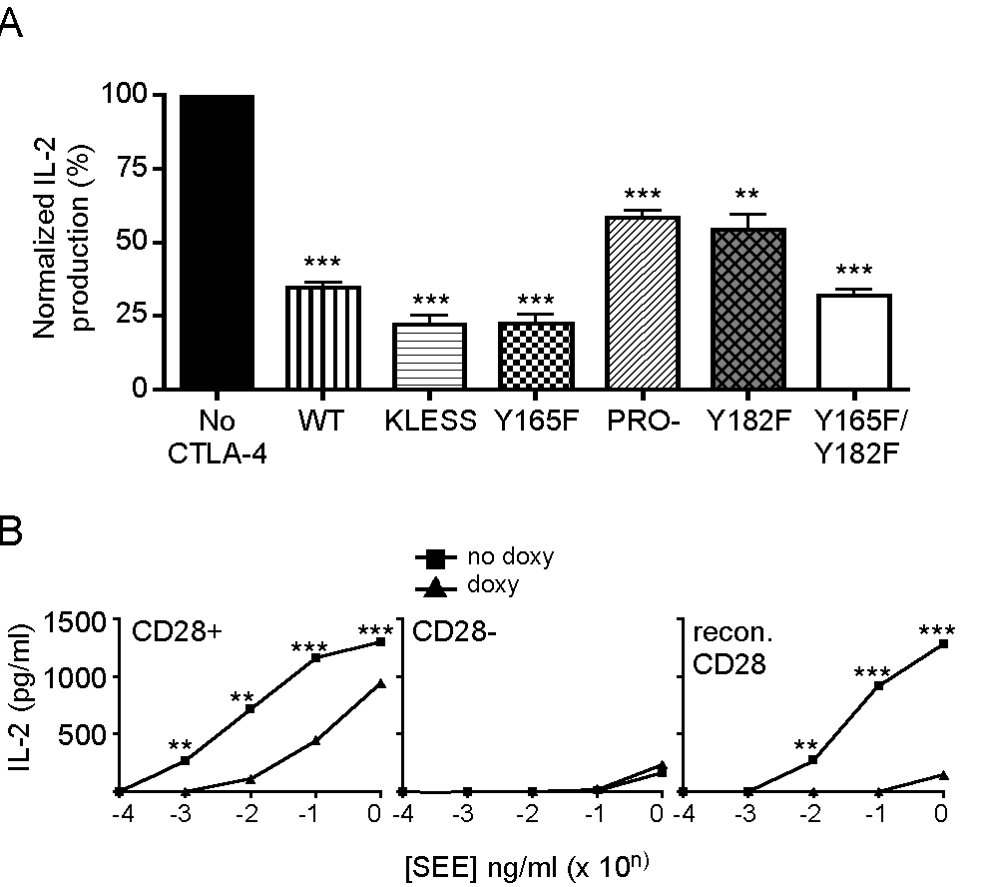
**Inhibitory function of CTLA-4 molecules with mutation in the cytoplasmic domain**. A) Jurkat T cells stably transfected with WT CTLA-4 or mutant CTLA-4 constructs were cultured overnight in the absence or presence of doxycycline (1 μg/ml). Cells were further stimulated with APCs and SEE (1 and 10 ng/ml) for 24 hours at 37°C. IL-2 production was measured by ELISA. Inhibition of IL-2 was determined for each CTLA-4 variant by calculating the percent of IL-2 produced in the presence of CTLA-4 expression normalized to the maximal IL-2 level in the absence of CTLA-4 at each concentration of SEE. The maximal level of inhibition was plotted for each CTLA-4 variant. This graph was generated using triplicate data for each concentration of SEE, from three independent experiments. B) CD28 expression is required for CTLA-4 mediated inhibition. CD28^+^, CD28^- ^and CD28-reconstituted Jurkat T cells were cultured overnight without (black squares) or with doxycycline (1 μg/ml) (black triangles) to induce the expression of Y165F CTLA-4. Cells were stimulated with SEE:APC and IL-2 was measured as in A). **, *p *< 0.01; ***, *p *< 0.001.

We have previously shown that PP2A plays a role as a negative regulator of the inhibitory function of CTLA-4, its primary function *in vivo *[10]. Therefore, we next determined the ability of each of the CTLA-4 variants examined above to attenuate T cell responses against the staphylococcal enterotoxin E (SEE) superantigen. Jurkat T cell lines containing the mutant CTLA-4 constructs were cultured overnight with or without doxycycline to induce the expression of CTLA-4 and were then stimulated in the presence of APC expressing B7 molecules on their surface and SEE at the indicated concentrations at 37°C for 24 hours (Figure 4A). The maximal level of inhibition was determined for each CTLA-4 variant by calculating the percent of IL-2 produced in the presence of CTLA-4 expression compared to cells stimulated in the absence of CTLA-4 induction. WT CTLA-4 as well as each mutant CTLA-4 was functionally able to inhibit T cell responses (Figure 4A). Cells expressing WT CTLA-4 attenuated IL-2 production in response to SEE stimulation by 65% on average. IL-2 production was inhibited between 68–78% by cells expressing CTLA-4 molecules with mutations in the lysine rich domain and the tyrosine 165 residue (KLESS, Y165F and Y165F/Y182F CTLA-4), while PRO- and Y182F CTLA-4 variants abrogated the response by 41% and 46%, respectively. The ability of each CTLA-4 variant to inhibit T cell responses suggests that the interaction between CTLA-4 and PP2A is not in itself the determinant of the inhibitory function of CTLA-4 and that there are additional factors that play a role in mediating this function.

One potential target of the interplay between CTLA-4 and PP2A may be CD28. As previously shown [15], we found that CD28 expression was required for CTLA-4-mediated inhibition (Figure 4B). CD28^+ ^or CD28^- ^Jurkat cells were cultured overnight with doxycycline to induce the expression of Y165F CTLA-4 and further stimulated with SEE:APC. IL-2 production was inhibited by CTLA-4 in cells expressing CD28. Such an inhibition of the response was not observed in cells lacking CD28 even though the amount of activation was significantly lower. However, reconstitution of CD28^- ^T cells expressing CTLA-4 with CD28 restored the inhibitory function of CTLA-4. This suggests that the inhibitory function of CTLA-4 requires the expression of CD28 indicating that CTLA-4 may likely act on the CD28 signaling pathway.

### The CTLA-4:PP2A interaction is required for the response to inverse agonists of CTLA-4

**Figure 5:**
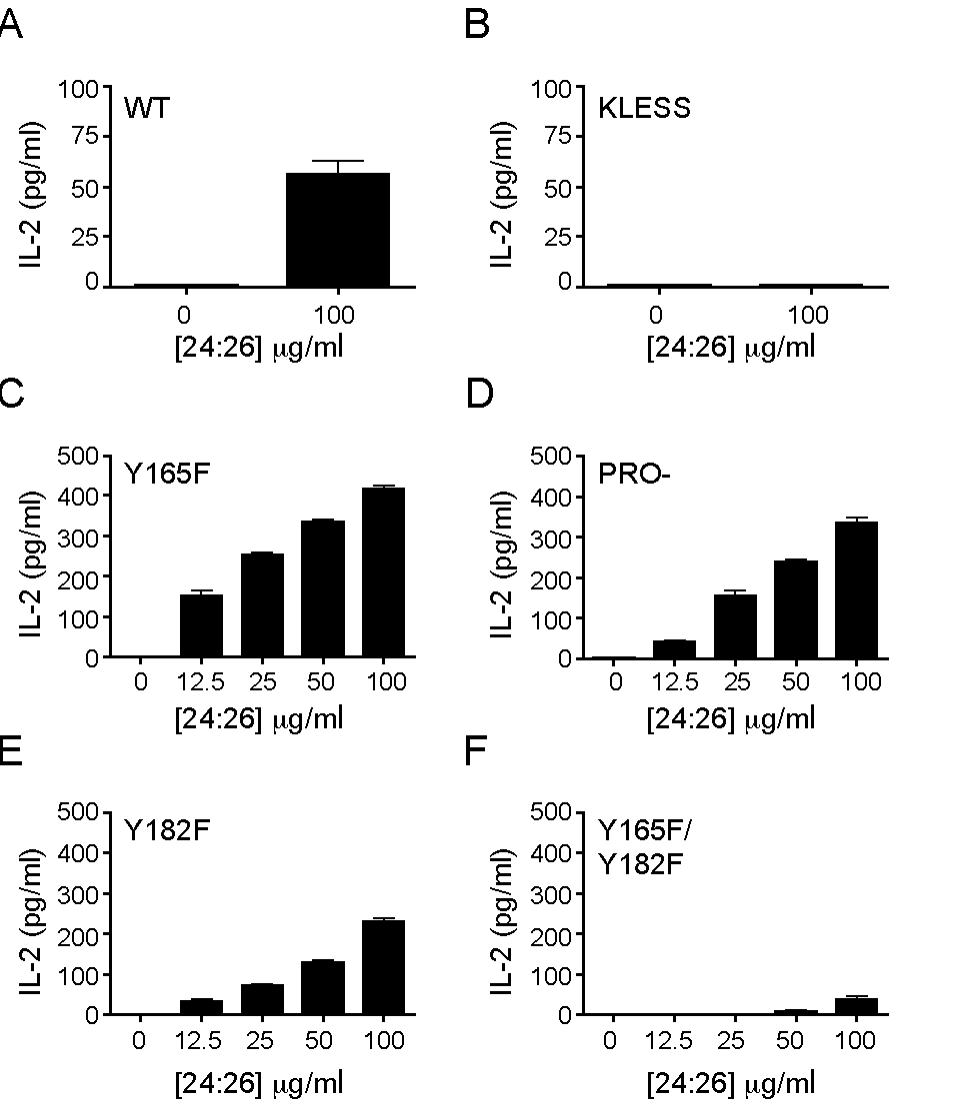
**The ability of CTLA-4 to activate T cells correlates with its interaction with PP2A**. Stably transfected Jurkat T cells were induced overnight with doxycycline (1 μg/ml) to express A) WT CTLA-4, B) KLESS CTLA-4, C) Y165F CTLA-4, D) PRO- CTLA-4, E) Y182F CTLA-4 or F) Y165F/Y182F CTLA-4. Cells were stimulated in triplicate in the presence of doxycycline with the indicated concentrations of 24:26 for 48 hours at 37°C. Supernatants were harvested and IL-2 was measured by ELISA. All data are representative of three independent experiments. **, *p *< 0.01; ***, *p *< 0.001.

Although the primary function of CTLA-4 is to inhibit T cell activation, we have recently shown that CTLA-4 has an inherent signaling plasticity. This plasticity is revealed by inverse agonists of CTLA-4 that can induce T cell activation by themselves in the absence of TCR engagement and CD28 costimulation [14,15]. As our panel of CTLA-4 variants contains mutations at key residues within the tail, it was ideal to determine the importance of these motifs in the response to inverse agonists of CTLA-4. To do this, we induced expression of CTLA-4 with doxycycline and stimulated the T cells with 24:26 at 37°C for 48 hours. IL-2 production was used as a readout for CTLA-4-dependent T cell activation (Figure 5). Engagement of WT CTLA-4 with 24:26 induced significant IL-2 production compared to unstimulated cells (Figure 5A). T cells expressing KLESS CTLA-4, a molecule that lacks association with PP2A, failed to respond to the inverse agonist 24:26, suggesting that PP2A binding to the lysine motif is important for the activating function of CTLA-4 (Figure 5B). The amount of IL-2 produced increased in a dose dependent manner upon ligation of PRO- CTLA-4, indicating that the proline residues are not essential for T cell activation by CTLA-4 inverse agonists (Figure 5D). 24:26 binding to Y165F CTLA-4 triggered a robust IL-2 response compared to the other CTLA-4 variants (Figure 5C). In contrast, CTLA-4 molecules mutated at Y182 showed a reduced ability to activate T cells when engaged with an inverse agonist compared to Y165F CTLA-4, suggesting that this residue may also be important for the activating function of CTLA-4 although less that the lysine-rich motif (Figure 5E). However, T cell activation by inverse agonists of CTLA-4 was practically abolished when both tyrosine residues were absent compared to CTLA-4 molecules mutated at either tyrosine residue alone, indicating that the both tyrosine residues may provide some functional redundancy with respect to inverse agonist activity (Figure 5F). Based on these biochemical and functional results, we concluded that the residues that are important for the association of PP2A to CTLA-4 are K152, 155 and156 and Y182, and that this association is required for the inverse agonist responses of CTLA-4.

### The association of PP2A to CTLA-4 is increased by inverse agonists of CTLA-4

**Figure 6:**
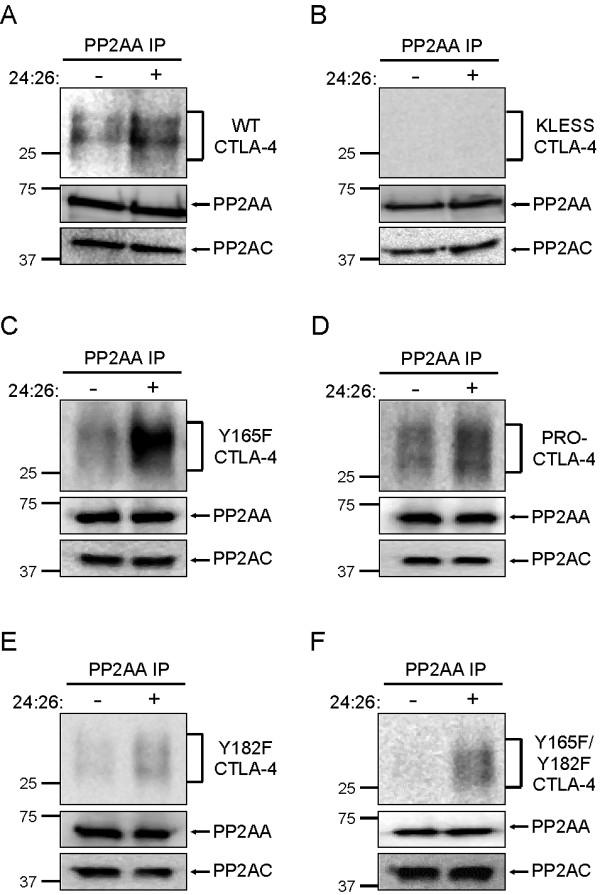
**24:26 increases the interaction of CTLA-4 with PP2A**. Stably transfected Jurkat T cells were induced overnight with doxycycline (1 μg/ml) to express A) WT CTLA-4, B) KLESS CTLA-4, C) Y165F CTLA-4, D) PRO- CTLA-4, E) Y182F CTLA-4 or F) Y165F/Y182F CTLA-4. The cells were stimulated with or without 24:26 (100 μg/ml) for 60 minutes at 37°C. Cells were washed then lysed in standard lysis buffer containing Triton X-100 (1%). Lysates were used for immunoprecipitation of PP2AA, and subsequently blotted for CTLA-4, PP2AA and PP2AC. All data is representative of at least two independent experiments.

We have previously reported that 24:26 binding to Y165F CTLA-4 stabilizes the association between PP2A and CTLA-4 [14]. Knowing the CTLA-4 residues involved in the interaction with PP2A allowed us to determine the molecular basis of such an increased association. To do this, we cultured the transfected T cell lines overnight in the presence of doxycycline to induce the expression of each CTLA-4 variant. T cells were then stimulated with 24:26 at 37°C for 60 minutes, lysed, immunoprecipitated with anti-PP2AA Abs and subsequently immunoblotted for CTLA-4. Immunoblotting for PP2AA and PP2AC was used as controls. We observed an increase in the amount of CTLA-4 co-precipitated with PP2AA following 24:26 engagement of WT CTLA-4 (Figure 6A), Y165F CTLA-4 (Figure 6C), and PRO- CTLA-4 (Figure 6D), correlating with the ability to induce T cell activation under similar stimulation conditions. In contrast, ligation of KLESS CTLA-4 with 24:26 was unable to induce co-precipitation with PP2AA (Figure 6B). Although little or no association was detected between PP2AA and Y182F CTLA-4 or Y165F/Y182F CTLA-4 in unstimulated conditions, a small increase in the level of association was seen upon inverse agonist ligation of these CTLA-4 molecules (Figure 6E, F). This low level of association correlated with the ability of Y182F CTLA-4 and Y165F/Y182F CTLA-4 to induce some IL-2 production when engaged with the inverse agonist 24:26, respectively.

### The inverse agonist properties of CTLA-4 are dependent on the phosphatase activity of PP2A

**Figure 7:**
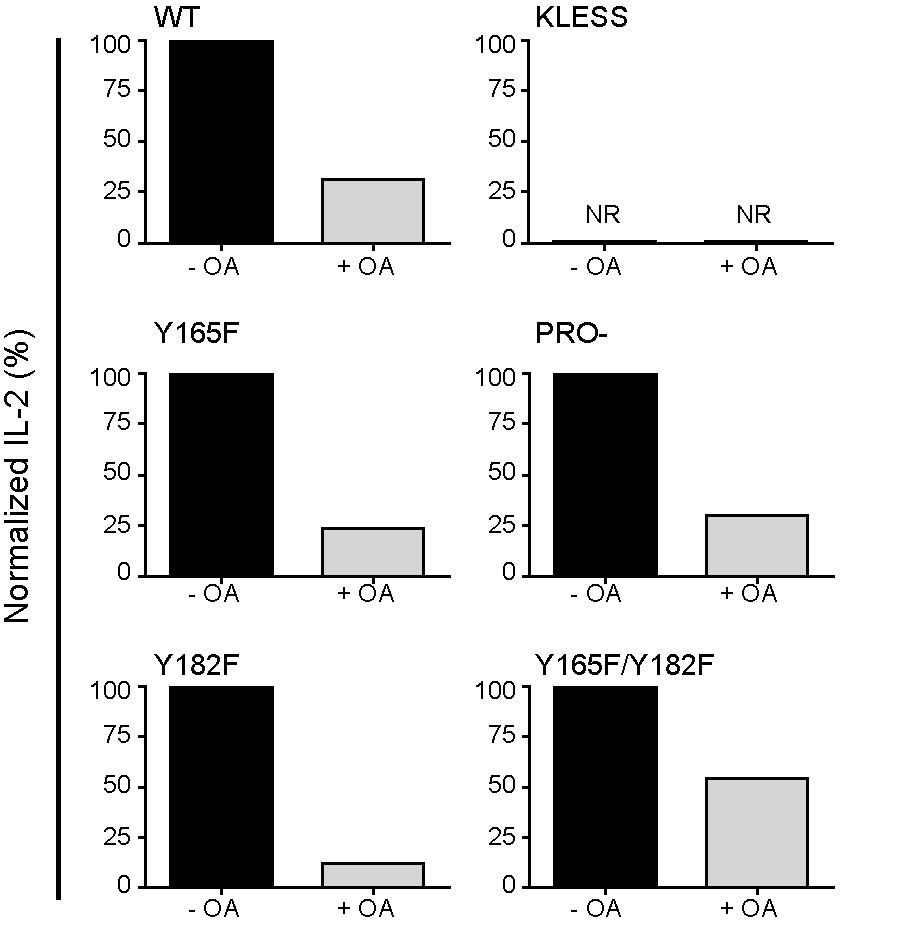
**The phosphatase activity of PP2A is required for CTLA-4-mediated T cell activation**. Stably transfected Jurkat T cells were cultured overnight in the presence of doxycycline (1 μg/ml) to induce the expression of WT CTLA-4 or CTLA-4 mutant molecules. The cells were further cultured in the presence of doxycycline and stimulated with 24:26 (100 μg/ml) in the presence or absence of OA (0.01 μM) for 48 hours at 37°C. Supernatants were harvested and IL-2 production was measured by ELISA. For each CTLA-4 variant the percent of IL-2 produced in the presence of OA was normalized to IL-2 levels in the absence of OA. All graphs are representative of at least two independent experiments.

We and others have shown that inhibition of T cell activation by CTLA-4 requires PP2A activity [10,13]. Specifically, CTLA-4 inhibits the activation of Akt, a molecule that is important in many cellular processes including IL-2 production [22]. CTLA-4-mediated inhibition of Akt activity is dependent on the phosphatase activity of PP2A as Akt phosphorylation was shown to be sensitive to the PP2A inhibitor okadaic acid (OA) [13]. Thus, we determined whether the activity of PP2A was important for the inverse agonist properties of CTLA-4. The panel of stably transfected Jurkat T cells was induced overnight in the presence of doxycycline to induce the expression of the CTLA-4 variants. The cells were further cultured with doxycycline and stimulated with 24:26 in the presence or absence of OA. IL-2 production was measured and normalized to the amount of IL-2 produced in the absence of OA for each of the CTLA-4 variants. We found that CTLA-4-mediated T cell activation was significantly inhibited by OA in T cells expressing WT and mutants forms of CTLA-4 excluding KLESS (Figure 7). Inhibition of inverse agonist activation by OA ranged from 45% (for Y165F/Y182F) to 88% (for Y182F) (69% for WT, 70% for PRO-, and 76% for Y165F). As shown above (Figure 5), KLESS CTLA-4 did not respond to 24:26 and thus no effect of OA was apparent (Figure 7). These results suggest that the phosphatase activity of PP2A is required for the inverse agonist response of CTLA-4.

## Discussion and conclusion

Understanding the mechanism of CTLA-4 function has proved to be remarkably puzzling over the past two decades. The ability of CTLA-4 to down-regulate T cell activation has been well established in multiple experimental systems including knock-out mouse models and T cell lines [4]. Both extrinsic and intrinsic factors contribute to the inhibitory mechanism of CTLA-4 *in vivo *[16]. Antagonism of CD28-dependent costimulation provides a plausible explanation for CTLA-4-mediated inhibition since CTLA-4 has a higher affinity and avidity for their shared ligands. However, the competition with CD28 for ligands only occurs when CTLA-4 is expressed at very high levels on the cell surface, indicating that an alternate mechanism lends to CTLA-4-dependent T cell inactivation [8]. The direct delivery of a negative signal provides a more likely explanation for the inhibitory function of CTLA-4 at early stages of T cell down-regulation. This mechanism is functional at low levels of CTLA-4 surface expression and requires an intact cytoplasmic domain. The precise signaling pathway initialized by CTLA-4 is still undefined although it has been linked to down-regulation of CD28-dependent events [23]. Many proteins have been shown to associate with CTLA-4. Among these, the serine/threonine phosphatase PP2A stands out as a candidate that can affect key molecules downstream of CD28, such as Akt, thereby affecting essential cellular events [24].

In this study, we dissected the interaction between PP2A and CTLA-4 both from a structural point of view, to identify the areas of interaction, as well as from a functional point of view, to establish the requirement of such an interaction for the inhibitory and activating effects of CTLA-4 ligation. This was done using a panel of Jurkat T cells stably transfected with WT CTLA-4 or CTLA-4 molecules mutated at various locations throughout the intracellular domain. Previous data from yeast two hybrid studies suggested that the cytoplasmic domain of mouse CTLA-4 interacted with two subunits of the core dimer of PP2A [9,10]. The core dimer is comprised of a scaffolding A subunit and a catalytic C subunit, each existing as α and β isoforms. The association of the dimer to a third regulatory B subunit provides the cellular localization and target specificity of PP2A [11]. Recent evidence has determined that post-translational modification of PP2AC plays an important role in the B subunit selection [11]. The requirements for the interaction of PP2A with CTLA-4 *in vivo *in human T cells were not identified, justifying the current study. We confirm here that the K-rich motif (located at lysine residues 152, 155 and 156) is required for the interaction of CTLA-4 with PP2A. Mutation of these residues to alanine (KLESS CTLA-4) abrogated CTLA-4:PP2A co-precipitation. This confirmed our previous observation under conditions of equalized expression of WT CTLA-4 and KLESS CTLA-4, suggesting that the lower expression of KLESS CTLA-4 in this study is not likely contributing to its lack of association with PP2A [10]. This observation is consistent with previous data pointing to the A subunit as the part of PP2A interacting with the K-rich motif [10].

The C subunit of PP2A was shown to associate biochemically with murine CTLA-4 in HEK293 cells transfected with the cytoplasmic domain of CTLA-4 fused to GST, and the interaction site was suggested to be the tyrosine 165 of the YVKM motif by yeast two hybrid analysis [9]. However, confirmation of the Y165 residue as the interaction site for PP2AC interaction was not examined in mouse or human T cells. An unexpected finding of our study here is that in human T cells it is the second tyrosine in the cytoplasmic tail of human CTLA-4 and not the first tyrosine (Y165) that is important for the interaction with PP2AC. We observed that co-precipitation of PP2A and CTLA-4 in human T cells was not affected when Y165 was mutated to phenylalanine. Since the A and C subunit of PP2A are almost always found associated with each other [12], this result suggested that either mutating the PP2AC binding site was not enough to break the CTLA-4:PP2AA interaction or that Y165 was not the essential residue. We found that both PP2AA and PP2AC were able to co-precipitate Y165F CTLA-4 molecules, implying that another residue was likely responsible for interacting with the C subunit of PP2A. Our data indicate that the key residue for the second site of the CTLA-4:PP2AA interaction in human T cells is Y182. CTLA-4 molecules mutated at this second tyrosine (Y182F CTLA-4) had severely diminished interaction with PP2A. We cannot exclude that the Y165 may contribute to this interaction when Y182 is not available because a small level of association is observed between Y182F CTLA-4 and PP2A. However, the same level of co-precipitation is noted when both tyrosine residues are mutated (Y165F/Y182F CTLA-4) indicating that Y182 is the important residue for binding PP2AC and that the small amount of association observed is likely due to the intact PP2AA binding motif. Based on our results we propose a model in which the CTLA-4:PP2A interaction occurs at two distinct binding motifs: one is the lysine-rich motif binding to the A subunit of PP2A and the other is the Y182 residue of CTLA-4 binding to the C subunit of PP2A. This model predicts that the lysine-rich motif is the primary site responsible for stabilizing the CTLA-4:PP2A interaction and the tyrosine residues may be less important since they may be redundant in their ability to interact with PP2AC. This prediction correlates with the functional data presented in this study.

From a functional point of view, CTLA-4 displays a remarkable plasticity as it can inhibit or even activate T cells depending on the ligand it engages and the conditions in which this engagement occurs. The primary physiological function of CTLA-4 is to down-regulate T cell activation. We have previously reported that, under conditions of TCR and CTLA-4 co-ligation, PP2A is phosphorylated and dissociates from CTLA-4 [10]. This correlates with the ability of CTLA-4 to inhibit T cell activation. This suggested that PP2A when bound to CTLA-4 prevents rather than mediates the inhibitory function of CTLA-4. In contrast, when PP2A is dissociated from CTLA-4, it likely inactivates downstream targets including Akt, consistent with the observation that CTLA-4-dependent inhibition of Akt phosphorylation is sensitive to OA [13]. This model is consistent with the findings reported here that all the CTLA-4 mutants, independently of their ability to bind PP2A, inhibited IL-2 production when co-ligated with the TCR. The magnitude of inhibition through CTLA-4 in different in vitro models, including our own, is relatively modest (50–70% on average) compared to the striking phenotype of CTLA-4 knockout mice. This may be due to the use of cell lines rather than primary cells, to more intense activation conditions used in the in vitro systems, or other factors. Still, such an inhibition is reproducible and statistically significant. It remains to be determined how such co-ligation of CTLA-4 and TCR triggers the activity of PP2A.

The other aspect of CTLA-4 function is its ability to activate T cells when binding recombinant inverse agonist ligands, such as soluble B7.1 Ig and 24:26. Under these conditions, PP2A also stands as a key player. We show here that all CTLA-4 variants capable of interacting with PP2A showed enhanced association with this phosphatase following CTLA-4 engagement with 24:26. Such an enhanced association is in contrast to the PP2A dissociation observed when CTLA-4 acts as an inhibitory receptor. This enhanced association between CTLA-4 and PP2A is likely the result of stabilization of the interaction between these two molecules. We have shown that 24:26 induces the formation of dimer-based CTLA-4 oligomers that are tightly associated with each other on the T cell surface [15]. The formation of such oligomers may provide a unique structure to facilitate the interaction between PP2A and CTLA-4. The enhanced CTLA-4:PP2A interaction upon inverse agonist ligation correlated with the ability of CTLA-4 to induce T cell activation. Moreover, the inverse agonist response was sensitive to the protein phosphatase inhibitor OA as IL-2 production induced upon 24:26 engagement of CTLA-4 was diminished its presence. Although OA is best known as an inhibitor of PP2A we can not rule out the inhibition of other phosphatases which may contribute to CTLA-4-mediated T cell activation. However, the effect of OA did not completely abolish the ability of CTLA-4 to induce IL-2 production, likely owing to the constitutive Akt activation in Jurkat T cells [25].

Delineation of the interaction between CTLA-4 and PP2A provides mechanistic insights into the signaling pathways targeted by CTLA-4. The costimulatory molecule CD28 has also been shown to associate with PP2A [9,10]. Microarray analysis of genes regulated upon B7 ligation of CTLA-4 suggests that CTLA-4 inhibits T cells by inhibiting CD28-dependent genes and not TCR-dependent genes [23]. Furthermore, we have shown that CD28 expression is essential for the inhibitory and activating function of CTLA-4 [15]. Therefore, it is plausible that CTLA-4 may function through PP2A as an inhibitor by blocking CD28 signaling and as an activator by triggering the CD28 signaling pathway. PP2A activity is dependent on its phosphorylation state, with unphosphorylated PP2A being active and phosphorylated PP2A rendered inactive. PP2A is able to dephosphorylate itself to regain activity [26].

One distinction between the inhibition of T cell activation by CTLA-4 and the activation of T cells by inverse agonists of CTLA-4 is that the former does not require the association of PP2A to CTLA-4 whereas the latter does. However, both responses are inhibited by okadaic acid [13], implying that PP2A is a critical mediator of them. We propose that, under conditions of T cell inhibition, PP2A is phosphorylated and dissociates from CTLA-4, becoming available to block CD28 signaling. Such a blockade may affect upstream events (eg., inhibition of lck, blockade of CD28-PI3K interaction), or downstream events (eg., direct inhibition of Akt). In this context, CTLA-4 would act as a shuttle of PP2A to the immunological synapse where, upon release, PP2A could act on TCR-ζ and CD28 signaling events [27]. It is unclear, how in our experimental system, CTLA-4 molecules that cannot bind PP2A can still inhibit T cell activation. Perhaps these molecules function to inhibit T cell responses primarily by sequestering B7 molecules from CD28.

Under conditions of T cell activation through CTLA-4, the pool of PP2A bound to CTLA-4 may trigger TCR-ζ and CD28 signaling by activating lck [14]. Lck activation, occurs through dephosphorylation of the negative regulatory tyrosine (residue 505) which induces autophosphorylation at tyrosine 394, initiating its kinase activity [28-30]. In addition to serine/threonine phosphatase acitivity, it has been reported that PP2A may also have tyrosine phosphatase activity [24]. Since both the phosphatase activity of PP2A and lck expression are required for 24:26-induced T cell activation [14], it is plausible to propose that PP2A could activate lck and initiate CD28 signaling.

## Methods

### Cells

Peripheral blood mononuclear cells (PBMC) were isolated from heparinized blood on Ficoll gradients (Amersham Pharmacia Biotech, Uppsala, Sweden). PBMC were cultured with 1 ng/ml PMA (Sigma-Aldrich, Oakville, Ontario, Canada) and 100 ng/ml ionomycin (Sigma-Aldrich) for 72 hours at 37°C, 5% CO_2 _to induce CTLA-4 expression. Cells were washed extensively, rested for 24 hours in fresh medium and used for biochemical experiments.

The stably transfected doxycycline-inducible CTLA-4 Jurkat T cell panel used for these studies has been previously described [10,14,17,27,31]. CD28^- ^cells stably expressing Y165F CTLA-4 were reconstituted with WT CD28. Stable transfectant clones were selected in the presence of G418. The B lymphoblastoid cell line, LG2, used as APC was provided by Dr. E. Long (National Institute of Allergy and Infectious Disease, National Institute of Health, Bethesda, MD). Cells were cultured in RPMI 1640 medium supplemented with 10% FCS.

### Antibodies and reagents

The mouse monoclonal antibody (Ab) 11 and the ScFv molecule 24:26, both against human CTLA-4 were generated at Wyeth Research (Cambridge, MA) and have been reported previously [4,10,14,17,27,31]. The following commercially available Abs were used in these studies: a goat polyclonal antiserum against the serine/threonine phosphatase 2A (PP2A) Aα (Santa Cruz Biotechnology, Santa Cruz, CA) and a mouse mAb against the PP2A catalytic subunit (Upstate Biotechnology, Lake Placid, NY. PE-labeled IgG2a were purchased from eBioscience (San Diego, CA). PE-labeled anti-human CTLA-4 was purchased from BD Biosciences (San Diego, CA). Staphylococcal enterotoxin E (SEE) was purchased from Toxin Technology (Sarasota, FL). Okadaic acid was purchased from Sigma-Aldrich (Oakville, Ontario, Canada).

### T cell functional assays

Doxycycline-induced Jurkat E6.1 T cell transfectants (0.1 × 10^6^/group) were cultured with or without SEE (1 ng/ml or 10 ng/ml)) or 24:26 at the concentrations indicated, and plated in triplicate in 96-well plates at 37°C for 24 or 48 hours, respectively [14]. Okadaic acid (0.01 μM) was added in the indicated experiments. IL-2 in culture supernatants was measured by ELISA (BD Biosciences).

### Flow cytometry

Stably transfected Jurkat T cells were cultured overnight with doxycycline (1 μg/ml) to induce the expression of CTLA-4. Cells (1 × 10^6^/group) were washed and stained with PE-labeled anti-CTLA-4 or isotype matched control on ice. Samples were then washed in PBS and analyzed by flow cytometry (Flowjo, Tree Star, Inc., Stanford University).

### Biochemistry

Doxycycline-induced Jurkat T cells (30 × 10^6^/group) were stimulated with or without 24:26 (100 μg/ml) at 37°C for 60 minutes. Primary human T cells (45 × 10^6^/group) were stimulated with PMA and ionomycin for 72 h, washed and further rested for 24 h. Cells were subsequently washed and lysed in standard lysis buffer containing Triton X-100 (1%). Cell lysates were immunoprecipitated with dithiobis succinimidyl propionate (DSP) cross-linked Abs on protein G agarose beads as previously described [17,32,33]. Protein samples were resolved by SDS-PAGE and analyzed by Western blotting using a digital image analyzer (Alpha Innotech).

### Statistics

Unpaired Student's *t *tests were performed using GraphPad Prism software. A difference between groups was considered significant when *p *≤ 0.05.

## Authors' contributions

WAT carried out the majority of the experimental work, participated in the experimental design and organization and drafted the manuscript. TAC performed some immunoprecipitation experiments. JM participated in the experimental design and organization and helped draft the manuscript. All authors have read and approved the manuscript for submission.
